# Tamoxifen in horses: pharmacokinetics and safety study

**DOI:** 10.1186/s13620-019-0143-7

**Published:** 2019-06-20

**Authors:** Gonzalo Gajardo, Rodrigo López-Muñoz, Anita Plaza, Benjamin Uberti, José Sarmiento, Gabriel Morán, Claudio Henríquez

**Affiliations:** 1 Escuela de Graduados, Facultad de Ciencias Veterinarias, Universidad Austral de Chile, Valdivia, Chile; 2 Instituto de Farmacología y Morfofisiología, Facultad de Ciencias Veterinarias, Universidad Austral de Chile, Valdivia, Chile; 3 Instituto de Medicina, Facultad de Medicina, Universidad Austral de Chile, Valdivia, Chile; 4 Instituto de Ciencias Clínicas, Facultad de Ciencias Veterinarias, Universidad Austral de Chile, Valdivia, Chile; 5 Instituto de Fisiología, Facultad de Medicina, Universidad Austral de Chile, Valdivia, Chile

**Keywords:** Tamoxifen, Pharmacokinetic, Horse, Safety

## Abstract

**Background:**

Tamoxifen (TAM), a selective modulator of estrogen receptors (SERMs) has been recently explored as a therapeutic option for the oral treatment of airway inflammation in the horse. The objective of this work was to establish pharmacokinetic parameters of TAM and its main metabolites in equines, as well as to determine its clinical safety in short-term treatments.

**Results:**

We determined TAM and its three main metabolites (4-OH tamoxifen, endoxifen, and N-desmethyl tamoxifen) in plasma after single administration of 0.25 mg/kg in healthy adult horses (*n* = 12). A maximum concentration of TAM was achieved 3 h after the oral administration (4.65 pg/mL ± 1.69); 4-OH tamoxifen was the metabolite that reached the highest concentration (78 pg/mL ± 70), followed by N-desmethyl tamoxifen (0.43 pg / mL ± 0.48), and finally endoxifen (0.17 pg/mL ± 0.17). All metabolites showed peak concentration 2 h after oral administration of the drug. Oral TAM bioavailability was 13,15% ± 4,18, with a steady state volume of distribution of 7831 ± 2922 (L/kg). Elimination half-life was 15.40 ± 5.80 h, and clearance was 5876 ± 699 (mL/kg/min). Clinical safety of TAM was determined over a 7-day course of treatment (0.25 mg/kg, orally q 24 h, *n* = 20). No adverse effects were observed through clinical examination, blood hematology, serum biochemistry, ophthalmological and reproductive examinations. Endometrial edema observed in some mares was attributed to normal cyclic activity.

**Conclusions:**

Tamoxifen has moderate oral bioavailability and a large volume of distribution, with three main metabolites in horses. Additionally, oral TAM administration over a 7-day treatment period demonstrated to be clinically safe, without adverse effects on clinical, hematological or serum biochemical parameters. These data could contribute to the continued research into this drug’s potential for the treatment of different inflammatory conditions in equine species.

**Electronic supplementary material:**

The online version of this article (10.1186/s13620-019-0143-7) contains supplementary material, which is available to authorized users.

## Introduction

Tamoxifen (TAM) is a nonsteroidal molecule derived from triphenylethylene, belonging to the group of selective modulators of estrogen receptors (SERMs) [1]. It was approved in 1977 by the Food and Drug Administration (FDA) for the treatment of advanced breast cancer, and is currently considered the main adjuvant for its treatment and prevention [2]. TAM has been shown to be metabolically activated in order to exert its antineoplastic effect [3, 4]. In humans, TAM is metabolized by enzymes of cytochrome P450 (CYP), resulting in the generation of different metabolites. N-desmethyltamoxifen (N-DMT) is produced by the action of CYP3A4 and CYP3A5 isoforms. N-DMT can be in turn transformed to 4-hydroxy-N-desmethyl-tamoxifen, also called endoxifen (EDF), by the action of CYP2D6. Another important metabolite is 4-hydroxy-tamoxifen (4-OH TAM), which is generated by the action of the CYP2D6 isoforms and to a lesser extent CYP3A4, CYP2B6 and CYP2C19 [5–7].

The term SERMs describes the fact that such molecules can act as agonists or antagonists depending on the target tissue. During its extensive use since its approval, TAM has proven to be a safe drug; however, different adverse effects have been described. Among the adverse effects reported in humans are nausea, gastrointestinal intolerance, hot flushes, bleeding or vaginal discharge, vulvar pruritus, rashes, dry skin, alopecia, headache, depression, confusion, fatigue, muscle cramps, increased incidence of deep vein thrombosis, pulmonary embolism, cataracts and endometrial cancer [8–10]. Additionally, it can compromise vision due to retinopathy, corneal opacity and cataracts [11–13]. In veterinary medicine, there is scarce information about adverse effects related to the use of TAM, with a single study on bitches reporting pyometra and retinitis [14].

Recently, TAM has been explored as a treatment for airway inflammation in horses [15], since it seems to induce a decrease in the neutrophilic infiltration and mucus accumulation in the airways. The aim of this work was to establish pharmacokinetic parameters of TAM and its main metabolites in equines, as well as to determine potential deleterious effects of its administration over a short course of treatment.

## Results

### Detection of TAM and its metabolites in plasma

**Fig. 1:**
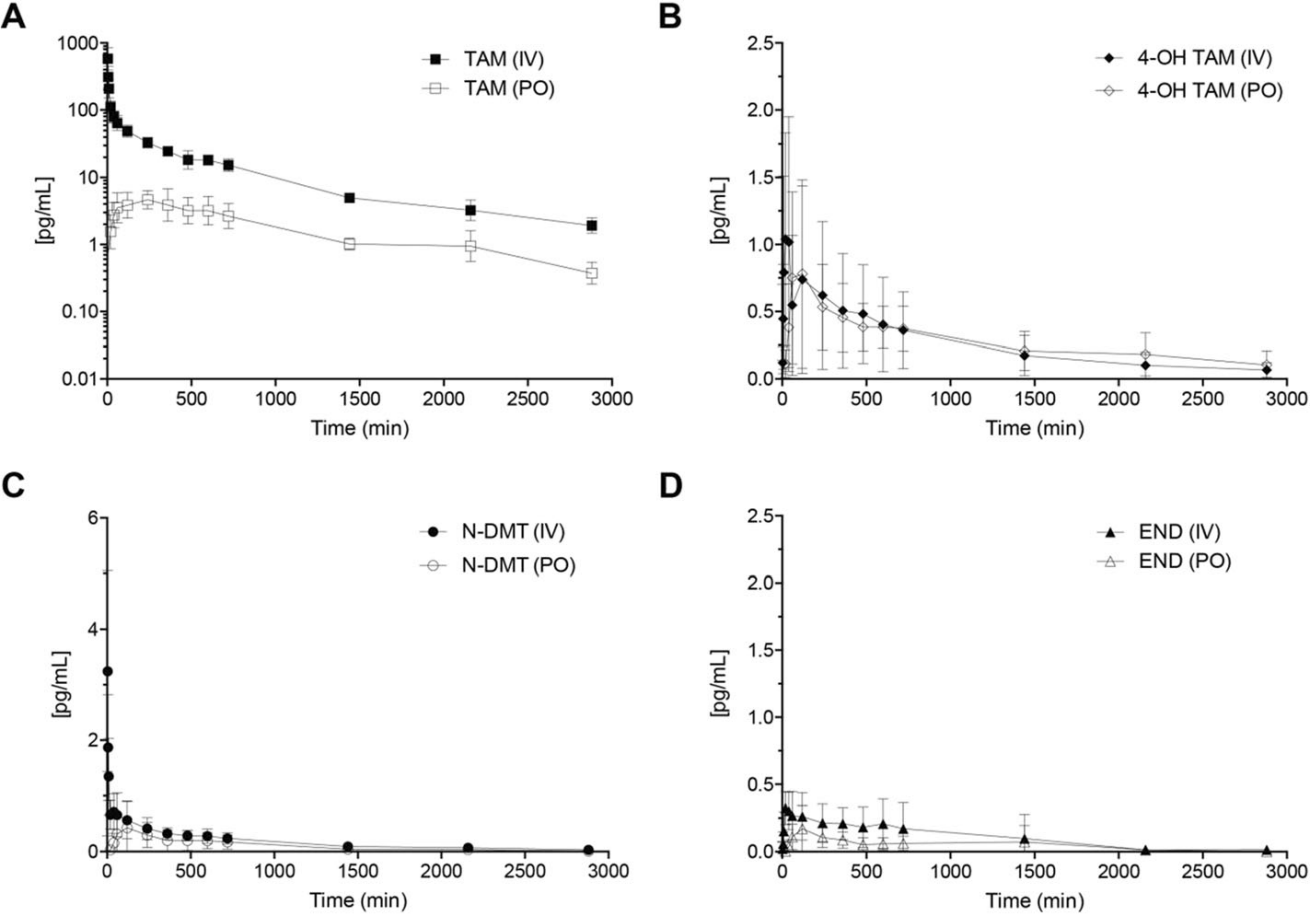
Plasmatic concentration of TAM and its metabolites, after single administration in healthy adult horses. **a** Plasmatic concentrations of TAM after intravenous (IV) and oral (PO) administration of a single dose of the drug (0.25 mg/kg); **b** Plasmatic concentrations of N-DMT after IV and PO administration; **c** 4-OH TAM, and **d** END (*n* = 6 for each route of administration)

**Fig. 2:**
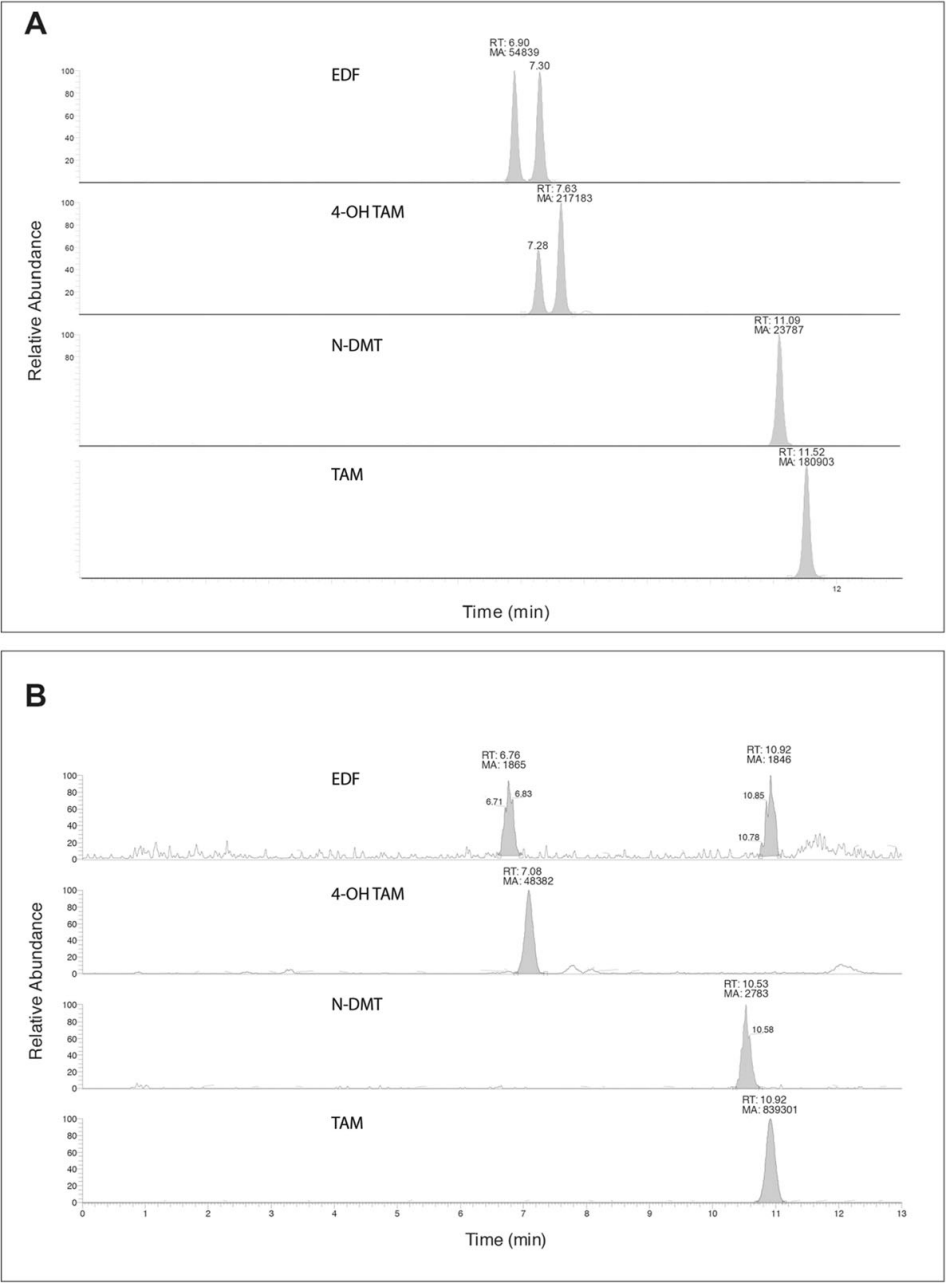
Chromatograms showing retention time of TAM and its three major metabolites. **a** Chromatograms of fortified plasma, showing the retention times of EDF, 4OH TAM, N-DMT and TAM, from top to bottom respectively. **b** Chromatograms of plasma samples obtained from treated adult healthy horses (0.25 mg/kg of body weight), showing the retention times of EDF, 4OH TAM, N-DMT and TAM, from top to bottom respectively

Ultra-performance liquid chromatography coupled to tandem MS was used to detect plasma TAM and the main metabolites described in other species. Figure 1a shows the chromatographic profile of a sample of the pool of high concentration (H) standards, indicating the retention times for endoxifen (EDF) isomers (6.90 min for E-EDF and 7.30 min for Z-EDF, respectively); there were two peaks for 4-OH TAM, with a retention time of 7.28 min and 7.63 min respectively. N-DMT had a retention time of 11.09 min, and TAM had the longest retention time, of 11.52 min. This allowed determination of the molecules’ plasma concentrations in treated horses (Fig. 1b), which yielded differences between fortified plasma and plasma from treated horses. For instance, only one peak for 4-OH TAM was present in all treated animals. There were two chromatography peaks for EDF in fortified plasma, one of which was absent in treated animals; a third peak appears later in the run, with a retention time of 10.9 min. EDF plasma concentration was estimated from both peak measurements, since the mass spectrum matched those from the molecule standards (Fig. 2).

### Pharmacokinetic parameters

Plasma concentration of TAM and its metabolites was determined by measuring the area of ​​the peaks for each molecule, and comparing each value obtained relative to the calibration curve. Figure 1a shows plasma concentrations of TAM after intravenous (IV) and oral (PO) administration. Additionally, Fig. 1b, c and d show plasma concentrations of all TAM metabolites (N-DMT, 4-OH TAM, and EDF respectively) after IV and PO administration. Pharmacokinetic parameters after the oral administration of a single dose of TAM were also determined. Maximum plasma TAM concentration varied between 3.3 and 9.6 ng/mL, between 1 and 6 h after administration. The elimination half-life also showed important inter-animal variation, ranging between 5.35 and 14.35 h (Table 1).

**Table 1:** Pharmacokinetic parameters of tamoxifen after single oral administration (0.25 mg/kg) in six adult healthy horses

	Animal						Mean	SD
	1	2	3	4	5	6		
AUC _0-∞_ (pg-mL/min)	5172.6	4069.4	4689.7	8974.0	4775.2	4624.9	5384.3	1793.9
C_max_ (pg/mL)	5.7	3.3	5.5	9.6	7.4	5	6.1	2.2
T_max_ (h)	1	4	4	6	2	1	3.0	2.0
t_1/2E_ (h)	10.24	13.53	14.35	5.35	7.13	12.5	10.5	3.6

In relation to TAM metabolites, the highest plasma concentration was reached by 4-OH TAM (0.78 pg/mL ± 0.70), followed by N-DMT (0.43 pg/mL ± 0.48) and finally END (0.17 pg/mL ± 0.17). Metabolite maximum plasma concentration time varied between 3 and 8 h (Table 2).

**Table 2:** Pharmacokinetic parameters of tamoxifen metabolites (N-DMT, 4-OH TAM, and EDF) after single oral administration of tamoxifen (0.25mg/kg) in adult healthy horses (n = 6)

	Metabolite		
	4-OH TAM	EDF	N-DMT
AUC _0-∞_ (pg-mL/min)	951.1 ± 461.9	249.1 ± 238.3	131.7 ± 107.6
C_max_ (pg/mL)	1.0 ± 0.6	0.5 ± 0.4	0.2 ± 0.1
T_max_ (h)	4.1 ± 4.2	3.0 ± 3.5	8.0 ± 8.8
t_1/2E_ (h)	9.8 ± 3.7	ND	ND

Pharmacokinetic parameters of TAM calculated from IV administration are presented in Table 3.

**Table 3:** Pharmacokinetic parameters of tamoxifen (mean ± standard deviation) after single intravenous administration (0.25 mg/kg) in healthy adult horses (n = 6)

Tamoxifen pharmacokinetic parameter	Result
Bioavailability (%)	13.15% ± 4.18
t_1/2E_ (h)	15.40 ± 5.80
Vss (L/Kg)	7831 ± 2922
Cl (ml/kg/min)	5876 ± 699

### Safety of TAM

Daily administration of TAM for 7 days showed no effects on respiratory and cardiac rate or on body temperature (Additional file 1: Figure S1). Likewise, there were no observed alterations in other parameters evaluated, such as hoof temperature, intestinal motility, or capillary refill time (data not shown). Erythrogram, leukogram and serum biochemistry showed no changes attributable to administration of the drug, neither by comparison with the control group nor in relation to reference values (Tables 4, 5 and 6).

**Table 4:** Erythrogram values before (Control) and after 4 (S1), 8 (S2), 11 (S3), and 15 (S4) days of treatment with tamoxifen (0.25 mg/kg, PO q 24 h for 7 days) in healthy adult horses (n = 20)

Sample	Control	S1	S2	S3	S4	Reference Range
Parameter	Mean ± SD					
Erythrocyte	7.71 ± 1.21	7.44 ± 1.03	7.49 ± 1.03	7.42 ± 0.94	7.32 ± 0.97	4.9–8.5 × 10^6 μL
Hematocrit	36 ± 0.05	36 ± 0.05	36 ± 0.05	36 ± 0.05	35 ± 0.04	27–42%
Hemoglobin	123 ± 19.53	120 ± 15.41	121 ± 16.48	119 ± 14.45	117 ± 14.15	94–147 g/L
MCV	45 ± 10.42	47 ± 2.15	48 ± 2.27	46 ± 11.80	47 ± 2.15	42–62 fL
MCHC	338 ± 21.12	339 ± 13.60	341 ± 25.55	316 ± 80.45	340 ± 12.60	320–380 g/L
Immature Eryth.	0 ± 0	0 ± 0	0 ± 0	0 ± 0	0 ± 0	0%
Platelets	152 ± 41.2	163 ± 48.8	159 ± 45.4	150 ± 46.8	153 ± 55.3	90-210 × 10^3^/ μL
Proteins	71 ± 4.03	72 ± 6.64	74 ± 5.76	72 ± 4.77	74 ± 3.85	68–84 g/L
Fibrinogen	3 ± 1.10	3 ± 2.90	3 ± 2.18	3 ± 0.81	3 ± 2.06	< 5 g/L

**Table 5:** Leukogram values before (Control) and after 4 (S1), 8 (S2), 11 (S3), and 15 (S4) days of treatment with tamoxifen (0.25 mg/kg, PO q 24 h for 7 days) in healthy adult horses (n = 20)

Sample	Control	S1	S2	S3	S4	Reference Range
Parameter	Mean ± SD					
Leukocytes	9443 ± 2595	9360 ± 2201	9310 ± 1958	8990 ± 1539	9225 ± 1761	7200–14,400 cel/μL
Basophils	46 ± 63.1	29 ± 71.85	26 ± 40.27	39 ± 50.92	54 ± 65.98	0–300 cel/μL
Eosinophils	467 ± 315.22	347 ± 275.49	406 ± 269.58	370 ± 328.23	259 ± 214.02	100–800 cel/μL
Neutrophils	4923 ± 1226	5232 ± 1341	5164 ± 1208	4862 ± 948.43	5108 ± 1388	2200–6100 cel/μL
Band neutrophils	0 ± 0	4 ± 16.77	0 ± 0	5 ± 21.24	0 ± 0	0–200 cel/μL
Juvenile neutrophils	0 ± 0	0 ± 0	0 ± 0	0 ± 0	0 ± 0	0 cel/μL
Lymphocytes	3395 ± 1169	3433 ± 1343	3406 ± 1455	3482 ± 1042	3475 ± 1455	1500–6500 cel/μL
Monocytes	202 ± 94.16	215 ± 116.37	151 ± 112.77	232 ± 159.13	211 ± 159.32	0–600 cel/μL

**Table 6:** Serum biochemistry before (Control) and after 4 (S1), 8 (S2), 11 (S3), and 15 (S4) days of treatment with tamoxifen (0.25 mg/kg, PO q 24 h for 7 days) in healthy adult horses (n = 20)

Sample	Control	S1	S2	S3	S4	Reference Range
Parameter	Mean ± SD					
Albumin	38 ± 4.27	38 ± 3.18	37 ± 5.99	38 ± 4.48	40 ± 5.61	26–38 g/L
AST	259 ± 97.57	243 ± 111.91	258 ± 138.37	272 ± 162.77	273 ± 96.96	140–480 U/L
Creatinine	96 ± 14.70	93 ± 79.17	102 ± 22.42	104 ± 31.86	98 ± 18.36	85–115 μmol/L
CK	332 ± 154.35	384 ± 306.83	263 ± 161.07	297 ± 166.16	276 ± 160.63	40–140 U/L
GGT	43 ± 43.03	40 ± 41.09	30 ± 14.08	32 ± 14.46	32 ± 20.73	12–62 U/L
SAP	585 ± 240.47	528 ± 244.99	549 ± 217.90	548 ± 266.68	557 ± 250.14	460-1060 U/L
LDH	621 ± 283.36	692 ± 192.79	659 ± 125.18	694 ± 121.91	663 ± 159.41	200–700 U/L
Total Proteins	74 ± 6.73	74 ± 5.66	76 ± 7.35	74 ± 6.52	77 ± 4.45	68–84 g/L
Urea	6.7 ± 1.39	7.4 ± 2.05	7.1 ± 1.24	7.2 ± 1.41	8.0 ± 2.16	3.6–8.8 mmol/L
Inorg. Phosphate	0.8 ± 0.28	0.8 ± 0.19	0.8 ± 0.23	0.9 ± 0.14	0.8 ± 0.28	0.9–1.5 mmol/L
Calcium	2.89 ± 0.23	2.92 ± 0.18	2.99 ± 0.27	2.85 ± 0.34	2.99 ± 0.21	2.49–3.21 mmol/L

Unlike previous reports in other species, no reproductive or ophthalmological adverse effects were observed after TAM administration, over a period of 7 days of treatment and 7 days of further follow-up. There were no differences in vulvar, vaginal or cervical characteristics before and after treatment. Three animals showed endometrial edema during the administration of the drug; however, this finding was normally present before the start of the treatment, and always correlated with normal reproductive cyclic activity and the presence of a pre-ovulatory follicle in one of the ovaries. There were no abnormal findings in any of the ophthalmological parameters evaluated (Additional file 2: Table S2).

## Discussion

In this study, we determined pharmacokinetic parameters of TAM in adult horses. The few clinical studies that have looked into the potential use of TAM for equine airway inflammation have been based on a single dosage (approximately 0,25 mg/kg) extrapolated from human medicine, with conflicting results regarding clinical efficacy based on that single empirical dose [15, 16]. However, there is robust pre-clinical information on modulation of inflammation, which warrants the pharmacokinetic and safety data presented here [17–21]. Oral TAM absorption was similar to findings in other species, since plasma concentration of TAM was maximum 3 h after oral administration, comparably to reports in humans and mice, where maximum concentration is also reached between 4 and 9 h post-administration [1]. In rats and dogs, peak plasma concentration varies between 5 to 22 h post-administration [22]. The bioavailability of TAM in the horses also proved to be similar to humans and rats, with a range between 15.8 and 32.8% described [23–27]. On the other hand, TAM presents a very high volume of distribution in the horse, several orders of magnitude higher to that in humans (52–62 L/kg) after repeated administration of the drug [28]. This result could have been influenced in part by the experimental intravenous formulation of TAM in DMSO, which may have affected its tissue distribution. The extraction process of our active drug from commercial oral formulations with talc-based excipients may have affected our calculations of pharmacokinetic parameters; however, this is unlikely due to the extreme solvent qualities of DMSO. This same caveat could also have affected its clearance, since the value obtained in this work exceeds previous reports in other species, of approximately 18 mL/min/kg [29–31]. The elimination half-life of TAM obtained in the present study was in the range described in humans, which varies from 9 to 49.2 h [32]. The elimination half-life is similar to that described in mice, rats, and humans (between 7 and 14 h), with a biphasic concentration curve and a terminal half-life of 7 days [32].

In relation to TAM metabolites, the highest plasma concentrations observed were those of 4-OH TAM, which is similar to what occurs in mice, whose concentrations of 4-OH TAM double those of other metabolites such as EDF. In humans, the predominant metabolite in plasma is N-DMT, followed by EDF and finally 4-OH TAM [33]. Similarly, in the rat the main metabolite is also N-DMT, which makes that species a better model for the study of pharmacokinetics in relation to humans. These differences could be due to CYP genetic polymorphisms, since in humans treated with TAM expressing an CYP2D6 isoform that metabolizes TAM poorly, the metabolite profile has been shown to be closer to mice than rats, with 4-OH TAM as the main metabolite [34, 35]. Horses express the CYP2D50 enzyme, an ortholog of CYP2D6, with 77% homology [36, 37], whose ability to metabolize drugs has also been shown to vary depending on which allele is inherited; this could partly explain the differences found with respect to humans.

TAM is a powerful therapeutic tool for the treatment of diseases in humans, such as breast cancer during all its stages. In this sense, the effect of this and other SERMs is mediated by their affinity for ERs. Other beneficial physiological effects have also been reported along with the positive effects of TAM in the treatment of cancer, including a decrease in circulating cholesterol, dissociation of fibrinogen and C-reactive protein, all of which are recognized as cardiovascular risk factors [38–41]. However, there are some relevant adverse effects described in long term treatments in people, such as increased risk of endometrial adenocarcinoma and uterine sarcoma [42–45]. As in humans, TAM has been proposed for veterinary use, specifically for the treatment of mammary adenocarcinoma in bitches [14]. However, the adverse effects associated with canine therapy seem to outweigh the benefits of its use. Among the main adverse effects described are an increase in vulvar flow, endometrial glandular hyperplasia, and pyometra. These effects occur quickly once treatment is established and cannot be prevented entirely through ovariohysterectomy. Unlike what has been described in the dog, in the present study TAM administration did not produce significant adverse effects in treated horses. Although minor reproductive differences were observed after treatment with TAM, specifically endometrial edema, these was always attributed to the reproductive cycle of each study subject.

Other adverse effects described in humans are related to ocular toxicity, including crystalline retinal deposits, macular edema, and corneal changes [11]. These findings are also described in other species were TAM has been used for the treatment of breast cancer, with retinitis reported in almost 50% of the treated dogs [14]. However, in our study, we found no ophthalmological lesions after 7 days of treatment and 7 days of further follow-up, either in cornea, crystalline or retina. This could be partly due to the lower dosage and plasma concentration in horses, compared to dogs and people.

Clinical and blood parameters were also monitored over a 7-day course of treatment, with no obvious adverse effects attributable to TAM. Different authors describe therapeutic potential of TAM on non-septic airway inflammation in horses, through induction of neutrophil early apoptosis in vitro and in vivo, resulting in resolution of inflammation [15, 17]. No neutropenia or effect on blood leukocyte populations was observed in those studies’ subjects. In humans neutropenia is a rare side effect of TAM, usually associated with thrombocytopenia and leukopenia, and only occurs in patients receiving high doses of the drug [46]. In this study, the absence of adverse drug reactions may well be associated to the low dose of TAM and relatively short course of treatment; however, most adverse effects in humans occur after long-term treatment [11, 46]. This suggests that TAM’s adverse effects may only appear after long-term treatment in equines. This is particularly relevant because equine asthma is a chronic disease, and long-term treatment with TAM may be of interest; thus, long-term pharmacokinetic data should be collected, and continuous monitoring of potential adverse drug reactions should be pursued over longer periods of time. Likewise, these experiments should be replicated in special populations, including pregnant mares and pediatric populations, rather than extrapolating the findings reported here.

## Conclusion

In this work, we determined plasma concentrations of TAM and its main metabolites in healthy adult horses, and calculated TAM pharmacokinetics, including bioavailability, half-life, volume of distribution, and clearance. TAM administration over a 7-day treatment period was clinically safe, without adverse effects on clinical, hematological or serum biochemical parameters.

## Materials and methods

### Animals

Thirty-two mixed breed adult horses, between 300 and 450 kg of live weight, were used in this study. The animals were maintained at Austral University of Chile’s Veterinary Teaching Hospital throughout the study, and belonged to its research herd. All animals were housed in individual stalls and fed mixed grass and alfalfa hay twice daily. The study was approved by the University’s Bioethics Committee (resolution N° 119, 2015).

All animals underwent complete clinical examinations, blood hematology and serum biochemistry analyses before inclusion in the study. Animals with any alteration were excluded. Of the 32 animals used, 12 took part in the pharmacokinetic study, and the remaining 20 animals took part in the safety study.

### Pharmacokinetic study

#### Equipment

Liquid chromatography was performed with an UltiMate™ 3000 RS system, coupled to a TSQ Vantage mass spectrometer (Thermo Fisher Scientific, USA). Separation was performed using a Syncronise C18 column of 50 mm length and 4.6 mm internal diameter, with a particle size of 1.7 μm (Thermo Fisher Scientific, USA).

#### Chemicals, reagents, and solutions

Standard molecules for quantification were TAM (Sigma-Aldrich), N-DMT (Sigma-Aldrich), 4-OH TAM (Sigma-Aldrich) and EDF (Tocris). All molecules were reconstituted according to the instructions of the laboratory of origin. For the elaboration of the calibration curves and evaluation of the extraction method, internal standards of each molecule were used, prepared in MeOH at 1 μM and kept at − 80 °C until their use.

The solvents used were HPLC grade acetonitrile (Merck Darmstadt, Germany), 98–100% formic acid (Merck Darmstadt, Germany), methanol (Merck Darmstadt, Germany) and ammonium formate (Fluka, Buchs, Switzerland). The mobile phase used for chromatography was composed of a 10 mM ammonium format in ultrapure water (solvent A) and acetonitrile (solvent B), both with 0.1% formic acid (FA) [47].

#### Conditions of the run

Determination of TAM and its metabolites was carried out replicating the determination protocol described by Dahmane et al [47], who proposes the use of a mobile phase gradient. The column was stabilized at 40 °C and the autosampler maintained at 4 °C. The mass spectrometry conditions used are shown in Additional file 3: Table S1.

#### TAM administration

In the present study, a single administration of 0.25 mg/kg of TAM was used. This dose was originally extrapolated from standard human dosages [48], and was the same dose used in previous equine clinical studies [15, 16]. The drug was administered after 8 h of overnight fasting.

For oral administration, tablets (Nolvadex 20 mg, Astra-Zeneca, London, UK) corresponding to each subject’s exact dose were crushed in a mortar. The resulting powder was resuspended in water, mixed with molasses and placed in a feeding syringe for subsequent oral administration; all administrations were done by one experienced clinician (BU), in order to avoid spillage. For intravenous administration, the same procedure described above was performed to obtain the powder. Subsequently, it was resuspended in 10 mL of DMSO and vigorously vortexed for 2 min. The suspension was stirred for 10 min and then centrifuged to remove talc from the tablet and recover the supernatant. The drug was administered after dilution in 0.9% NaCl and filtration with a 0.22 μm syringe filter, through aseptic injection in the right jugular vein.

#### Blood sample collection

Blood samples for determination of drug concentrations were obtained by left jugular venipuncture and collected in 4 mL tubes, using EDTA as an anticoagulant. The blood samples were obtained at 0; 20; 40; 60; 120; 240; 360; 480; 600; 720; 1440; 2160 and 2880 min after oral or intravenous administration.

#### Extraction process

Plasma samples obtained from treated horses were aliquoted in 100 μL and stored in plastic microtubes at − 80 °C until processing. For the extracting procedure, 100 μL of plasma was mixed with 300 μL of acetonitrile and vortexed for 10 s, and then sonicated for 30 s. The sample was then centrifuged at 4 °C for 10 min at 16,000 *g* and the resulting supernatant transferred to 1.5 mL tubes. The samples were evaporated to dryness in SpeedVac® for 120 min at room temperature. The resulting solid residue was reconstituted with 60 μL of a solution of MeOH / 20 mM ammonium formate 1:1 (v/v), adjusted to pH 2.9 with formic acid. It was mixed using vortex, and the sample was subsequently centrifuged under the same conditions. The supernatant was placed in 200 μL inserts, inside 1.5 mL glass microvials, and in turn positioned in the autosampler and maintained at 4 °C during the LC-MS/MS analysis. The injection volume used was 30 μL.

#### Matrix effect, extraction performance, and recovery

For the determination of these variables, blank plasmas (obtained from 6 animals) were fortified with TAM or each of its metabolites, in order to determine the absence of interferences in the chromatographic reading, retention times for each one of the analytes and perform the corresponding mass analysis.

The determination of the matrix effect was carried out by processing three series of samples using the stocks of TAM and its metabolites. First, a pool of standards at a concentration of 1 μM in MeOH was prepared, to then perform the dilutions of said pool in blank plasma, until obtaining 3 concentrations. These concentrations were defined as high or H (0.02 μM), medium or M (0.0025 μM) and low or L (0.0003125 μM). The series of samples were made in the order:

- The pool of standards (concentrations H, M, and L) was dissolved in MeOH with NH_4_HCO_2_ at 20 mM (adjusted to pH 2.9 with formic acid).
- Plasma obtained from 6 different specimens, fortified with the pool of standards in its different concentrations (concentrations H, M, and L), after the extraction process.
- Plasma obtained from 6 different specimens, fortified with the pool of standards in its different concentrations (concentrations H, M, and L), prior to the extraction process.

Subsequently, the recovery and suppression or improvement of the signal of the analytes in the presence of the matrix were evaluated by comparing the areas of the absolute peaks obtained between the A and B series dissolved in reconstitution solvent.

The yield of the extraction was calculated by establishing the percentage ratio between the fortified plasma with the stock solution, prior to the extraction process (C), expressed as a percentage with respect of the same amount of analyte added to the plasma after the extraction procedure (B).

#### Calibration curve and limit of determination

The analysis of TAM, NDT, HTF, and EDF in plasma was performed using a method of internal standards. For this purpose, plasma obtained from untreated, healthy animals was fortified with TAM and their metabolites in concentrations of 0.02, 0.01, 0.005, 0.003, 0.001, 0.0005 and 0.0003 μM. The calibration curves were calculated and adjusted by a logarithmic quadratic regression establishing the ratio of the area of each peak of TAM, NDT, HTF and EDF [47]. The lower limit of quantification was determined by fortifying the plasma with the molecules of TAM, NDT, HTF, and EDF, in concentrations of 0.1, 1, 10, 100 and 1000 ng / mL.

### Pharmacokinetic parameters

Pharmacokinetic parameters were calculated using the software PK Solutions version 2.0 (Summit Research Services, Montrose, Colorado, USA), an Excel based software used in other pharmacokinetic studies [49, 50]. The parameters determined were AUC_0-∞_, C_max_, T_max_, t_1/2E_, bioavailability, Vss and Cl.

### Safety study of TAM

**Fig. 3:**
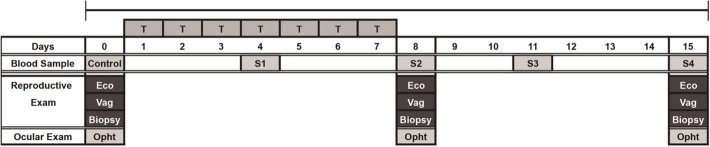
Timeline for the safety study of TAM administration in horses. Oral administration of TAM was done every day for seven days (0.25 mg/kg of body weight) in healthy adult horses. T = treatment; S = blood sampling; Eco = ecographic examination; Vag = reproductive examination; Opht = ophthalmological examination

For the safety study, 20 clinically healthy animals (8 geldings and 12 mares) received a TAM dose of 0.25 mg/kg of body weight, orally, once daily for 7 consecutive days, with a follow-up period of 7 days as described in Fig. 3.

### Physical examination

General clinical examinations were carried out on each horse for inclusion in the study, excluding subjects with any alteration. After inclusion, all study subjects underwent daily clinical evaluations, including heart and respiratory rates, rectal temperature, intestinal motility, capillary refill time, hoof temperature, digital pulse, consumption of water and food intake, production and consistency of feces.

### Blood parameters

Blood hematology and serum biochemistry was performed on samples from all study subjects, prior to inclusion in the study, in order to exclude patients with any alteration. The same analyses were repeated before (day 0), during (day 4) and after (days 8, 11 and 15) the period of drug administration (Fig. 3 and Tables 2, 3, 4).

### Examination of the reproductive system

Evaluation of the vulva, vagina, uterus, uterine horns and ovaries was carried out through transrectal palpation and ultrasound (Mindray Dp-50, Shenzhen, China) with transrectal 5–10 MHz linear transducer on days 0, 8 and 15 of the study.

### Ophthalmological examination

All study subjects underwent bilateral opthalmological examination on days 0, 8 and 15 of the study (Fig. 3). This was performed by an experienced clinician, in standing stocks, with local anesthesia of the auriculopalpebral nerve with 2 mL of 2% lidocaine solution. The injection point was located on the dorsal aspect of the zygomatic arch, at the midpoint between the lateral canthus of the eye and the base of the ear [51]. Midriasis was induced by topical application of 4 drops of 1% Tropicamide (Mydriacil®) per eye. Ocular fundus examination was done according to previous descriptions, using the Camera Awesome application (© SmugMug Inc.) for iPhone 4 (Apple, California USA) [52].

### Statistical analysis

The results obtained from the quantitative variables are presented as arithmetic means and standard deviation (SD). The distribution of the data was evaluated using the Shapiro Wilk test, while the repeated measurements ANOVA test was used to compare the different samples. *P*-values < 0.05 were considered statistically significant.

## Additional files

Additional file 1: **Figure S1.** Clinical examination findings in healthy adult horses treated with tamoxifen. (DOCX 1020 kb)

Additional file 2: **Table S2.** Ophthalmological findings in healthy adult horses treated with tamoxifen. (PDF 30 kb)

Additional file 3: **Table S1.** LC–MS/MS conditions used for determination of TAM and its metabolites in equine plasma. (PDF 29 kb)

## Data Availability

All raw data related to this work will be available to researchers who may request them from the corresponding author.

## Abbreviations

4-OH TAM: 4-OH Tamoxifen
AUC_0-∞_: Area under the curve from 0 h to infinity
C_max_: Peak plasma concentration
DMSO: Dimethyl Sulfoxide
EDF: Endoxifen
EDTA: Ethylenediaminetetraacetic acid
ERs: Estrogen receptors
MeOH: Methanol
N-DMT: N-Desmethyltamoxifen
NH_4_HCO_2_: Ammonium formate
SERMs: Selective estrogen receptors modulators
t_1/2E_: Elimination half-life
TAM: Tamoxifen
T_max_: Time to reach C_max_

## References

[1] Jordan VC (2007). New insights into the metabolism of tamoxifen and its role in the treatment and prevention of breast cancer. Steroids.

[2] Cuzick J, Sestak I, Cawthorn S, Hamed H, Holli K, Howell A, Forbes JF, Investigators I-I (2015). Tamoxifen for prevention of breast cancer: extended long-term follow-up of the IBIS-I breast cancer prevention trial. Lancet Oncol.

[3] Maximov PY, Lee TM, Jordan VC (2013). The discovery and development of selective estrogen receptor modulators (SERMs) for clinical practice. Curr Clin Pharmacol.

[4] Teunissen SF, Rosing H, Schinkel AH, Schellens JH, Beijnen JH (2010). Bioanalytical methods for determination of tamoxifen and its phase I metabolites: a review. Anal Chim Acta.

[5] Antunes MV, Rosa DD, Viana Tdos S, Andreolla H, Linden R, Fontanive TO (2013). Sensitive HPLC-PDA determination of tamoxifen and its metabolites N-desmethyltamoxifen, 4-hydroxytamoxifen and endoxifen in human plasma. J Pharm Biomed Anal.

[6] Antunes MV, Raymundo S, de Oliveira V, Staudt DE, Gossling G, Peteffi GP, Biazus JV, Cavalheiro JA, Tre-Hardy M, Capron A (2015). Ultra-high performance liquid chromatography tandem mass spectrometric method for the determination of tamoxifen, N-desmethyltamoxifen, 4-hydroxytamoxifen and endoxifen in dried blood spots--development, validation and clinical application during breast cancer adjuvant therapy. Talanta.

[7] Dickschen K, Willmann S, Thelen K, Lippert J, Hempel G, Eissing T (2012). Physiologically based pharmacokinetic modeling of tamoxifen and its metabolites in women of different CYP2D6 phenotypes provides new insight into the tamoxifen mass balance. Front Pharmacol.

[8] Swerdlow AJ, Jones ME (2005). British tamoxifen second Cancer study G: tamoxifen treatment for breast cancer and risk of endometrial cancer: a case-control study. J Natl Cancer Inst.

[9] Smith LL, Brown K, Carthew P, Lim CK, Martin EA, Styles J, White IN (2000). Chemoprevention of breast cancer by tamoxifen: risks and opportunities. Crit Rev Toxicol.

[10] Brown K (2002). Breast cancer chemoprevention: risk-benefit effects of the antioestrogen tamoxifen. Expert Opin Drug Saf.

[11] Nayfield SG, Gorin MB (1996). Tamoxifen-associated eye disease. A review. J Clin Oncol.

[12] Bourla DH, Sarraf D, Schwartz SD (2007). Peripheral retinopathy and maculopathy in high-dose tamoxifen therapy. Am J Ophthalmol.

[13] Kim LA, Amarnani D, Gnanaguru G, Tseng WA, Vavvas DG, D'Amore PA (2014). Tamoxifen toxicity in cultured retinal pigment epithelial cells is mediated by concurrent regulated cell death mechanisms. Invest Ophthalmol Vis Sci.

[14] Tavares WL, Lavalle GE, Figueiredo MS, Souza AG, Bertagnolli AC, Viana FA, Paes PR, Carneiro RA, Cavalcanti GA, Melo MM (2010). Evaluation of adverse effects in tamoxifen exposed healthy female dogs. Acta Vet Scand.

[15] Perez B, Henriquez C, Sarmiento J, Morales N, Folch H, Galesio JS, Uberti B, Moran G (2016). Tamoxifen as a new therapeutic tool for neutrophilic lung inflammation. Respirology.

[16] Mainguy-Seers S, Picotte K, Lavoie JP (2018). Efficacy of tamoxifen for the treatment of severe equine asthma. J Vet Intern Med.

[17] Sarmiento J, Perez B, Morales N, Henriquez C, Vidal L, Folch H, Galecio JS, Moran G (2013). Apoptotic effects of tamoxifen on leukocytes from horse peripheral blood and bronchoalveolar lavage fluid. Vet Res Commun.

[18] Borlone C, Morales N, Henriquez C, Folch H, Olave C, Sarmiento J, Uberti B, Moran G (2017). In vitro effects of tamoxifen on equine neutrophils. Res Vet Sci.

[19] Morales N, Henriquez C, Sarmiento J, Uberti B, Moran G (2018). Tamoxifen inhibits chemokinesis in equine neutrophils. Ir Vet J.

[20] Olave C, Alvarez P, Uberti B, Morales N, Henriquez C, Folch H, Sarmiento J, Moran G (2019). Tamoxifen induces apoptosis and inhibits respiratory burst in equine neutrophils independently of estrogen receptors. J Vet Pharmacol Ther.

[21] Olave C, Morales N, Uberti B, Henriquez C, Sarmiento J, Ortloff A, Folch H, Moran G (2018). Tamoxifen induces apoptotic neutrophil efferocytosis in horses. Vet Res Commun.

[22] Fromson JM, Pearson S, Bramah S (1973). The metabolism of tamoxifen (I.C.I. 46,474). I. in laboratory animals. Xenobiotica.

[23] Shin SC, Choi JS, Li X (2006). Enhanced bioavailability of tamoxifen after oral administration of tamoxifen with quercetin in rats. Int J Pharm.

[24] Kim CS, Choi SJ, Park CY, Li C, Choi JS (2010). Effects of silybinin on the pharmacokinetics of tamoxifen and its active metabolite, 4-hydroxytamoxifen in rats. Anticancer Res.

[25] Li C, Lim SC, Kim J, Choi JS (2011). Effects of myricetin, an anticancer compound, on the bioavailability and pharmacokinetics of tamoxifen and its main metabolite, 4-hydroxytamoxifen, in rats. Eur J Drug Metab Pharmacokinet.

[26] Tukker JJ, Blankenstein MA, Nortier JW (1986). Comparison of bioavailability in man of tamoxifen after oral and rectal administration. J Pharm Pharmacol.

[27] Choi JS, Kang KW (2008). Enhanced tamoxifen bioavailability after oral administration of tamoxifen in rats pretreated with naringin. Arch Pharm Res.

[28] Lien EA, Solheim E, Lea OA, Lundgren S, Kvinnsland S, Ueland PM (1989). Distribution of 4-hydroxy-N-desmethyltamoxifen and other tamoxifen metabolites in human biological fluids during tamoxifen treatment. Cancer Res.

[29] Shin SC, Piao YJ, Choi JS (2008). Effects of morin on the bioavailability of tamoxifen and its main metabolite, 4-hydroxytamoxifen, in rats. In Vivo.

[30] Piao Y, Shin SC, Choi JS (2008). Effects of oral kaempferol on the pharmacokinetics of tamoxifen and one of its metabolites, 4-hydroxytamoxifen, after oral administration of tamoxifen to rats. Biopharm Drug Dispos.

[31] Cho YA, Lee W, Choi JS (2012). Effects of curcumin on the pharmacokinetics of tamoxifen and its active metabolite, 4-hydroxytamoxifen, in rats: possible role of CYP3A4 and P-glycoprotein inhibition by curcumin. Pharmazie.

[32] de Vos D, Slee PH, Stevenson D, Briggs RJ (1992). Serum elimination half-life of tamoxifen and its metabolites in patients with advanced breast cancer. Cancer Chemother Pharmacol.

[33] Woo HI, Lee SK, Kim J, Kim SW, Yu J, Bae SY, Lee JE, Nam SJ, Lee SY (2017). Variations in plasma concentrations of tamoxifen metabolites and the effects of genetic polymorphisms on tamoxifen metabolism in Korean patients with breast cancer. Oncotarget.

[34] Borges S, Desta Z, Li L, Skaar TC, Ward BA, Nguyen A, Jin Y, Storniolo AM, Nikoloff DM, Wu L (2006). Quantitative effect of CYP2D6 genotype and inhibitors on tamoxifen metabolism: implication for optimization of breast cancer treatment. Clin Pharmacol Ther.

[35] Jin Y, Desta Z, Stearns V, Ward B, Ho H, Lee KH, Skaar T, Storniolo AM, Li L, Araba A (2005). CYP2D6 genotype, antidepressant use, and tamoxifen metabolism during adjuvant breast cancer treatment. J Natl Cancer Inst.

[36] DiMaio Knych HK, Stanley SD (2008). Complementary DNA cloning, functional expression and characterization of a novel cytochrome P450, CYP2D50, from equine liver. Biochem Pharmacol.

[37] Corado CR, McKemie DS, Young A, Knych HK (2016). Evidence for polymorphism in the cytochrome P450 2D50 gene in horses. J Vet Pharmacol Ther.

[38] Gundimeda U, Chen ZH, Gopalakrishna R (1996). Tamoxifen modulates protein kinase C via oxidative stress in estrogen receptor-negative breast cancer cells. J Biol Chem.

[39] Jordan VC (2006). The science of selective estrogen receptor modulators: concept to clinical practice. Clin Cancer Res.

[40] Birzniece V, Barrett PHR, Ho KKY (2017). Tamoxifen reduces hepatic VLDL production and GH secretion in women: a possible mechanism for steatosis development. Eur J Endocrinol.

[41] Kmietowicz Z (2014). Tamoxifen reduces breast cancer rate in at-risk healthy women by nearly a third, finds study. BMJ.

[42] Lavie O, Barnett-Griness O, Narod SA, Rennert G (2008). The risk of developing uterine sarcoma after tamoxifen use. Int J Gynecol Cancer.

[43] Wickerham DL, Fisher B, Wolmark N, Bryant J, Costantino J, Bernstein L, Runowicz CD (2002). Association of tamoxifen and uterine sarcoma. J Clin Oncol.

[44] Matsuo K, Ross MS, Bush SH, Yunokawa M, Blake EA, Takano T, Ueda Y, Baba T, Satoh S, Shida M (2017). Tumor characteristics and survival outcomes of women with tamoxifen-related uterine carcinosarcoma. Gynecol Oncol.

[45] Felix AS, Cook LS, Gaudet MM, Rohan TE, Schouten LJ, Setiawan VW, Wise LA, Anderson KE, Bernstein L, De Vivo I (2013). The etiology of uterine sarcomas: a pooled analysis of the epidemiology of endometrial cancer consortium. Br J Cancer.

[46] Mike V, Currie VE, Gee TS (1994). Fatal neutropenia associated with long-term tamoxifen therapy. Lancet.

[47] Dahmane E, Mercier T, Zanolari B, Cruchon S, Guignard N, Buclin T, Leyvraz S, Zaman K, Csajka C, Decosterd LA (2010). An ultra performance liquid chromatography-tandem MS assay for tamoxifen metabolites profiling in plasma: first evidence of 4′-hydroxylated metabolites in breast cancer patients. J Chromatogr B Analyt Technol Biomed Life Sci.

[48] Lazzeroni M, Serrano D, Dunn BK, Heckman-Stoddard BM, Lee O, Khan S, Decensi A (2012). Oral low dose and topical tamoxifen for breast cancer prevention: modern approaches for an old drug. Breast Cancer Res.

[49] Charles BG, Duffull SB (2001). Pharmacokinetic software for the health sciences: choosing the right package for teaching purposes. Clin Pharmacokinet.

[50] Nascimento JW, Carmona MJ, Strabelli TM, Auler JO, Santos SR (2007). Perioperative cefuroxime pharmacokinetics in cardiac surgery. Clinics (Sao Paulo).

[51] SJ MW, JS WM (2011). Regional Anesthesia: Nerve blocks of the head. Equine Joint Injection and Regional Anesthesia..

[52] Brooks DE (2014). How to use a smartphone camera for ocular photography in the horse. Ophthalmology: clinical examination, photographic documentation, and treatment options. 60th Annual AAEP Convention: 2014.

