# Supplementary material for: Tamoxifen in horses: pharmacokinetics and safety study
Source: Ir Vet J. 2019 Jun 20;72:5. doi: 10.1186/s13620-019-0143-7 (PMC6587269; doi:10.1186/s13620-019-0143-7)
Supplement: Supplementary file 1 — Figure S1. Clinical examination findings in healthy adult horses treated with tamoxifen. (DOCX 1020 kb) [file 13620_2019_143_MOESM1_ESM.docx]

**
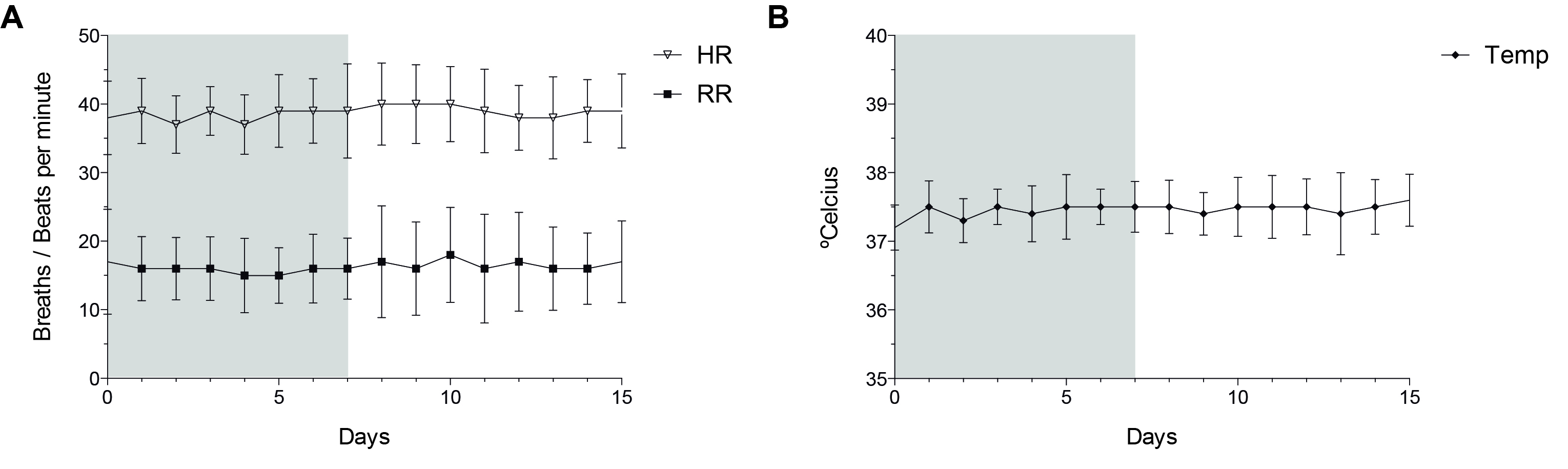
Additional file 1: Figure S1**  Clinical examination findings in healthy adult horses treated with tamoxifen (0.25 mg/kg, orally, q 24 hours for 7 days) (gray area, days 0-7), with 7 days of follow-up after the treatment period (days 8-15) (n=20). A) Respiratory and heart rates; B) Rectal temperature.
