# Supplementary material for: Tamoxifen in horses: pharmacokinetics and safety study
Source: Ir Vet J. 2019 Jun 20;72:5. doi: 10.1186/s13620-019-0143-7 (PMC6587269; doi:10.1186/s13620-019-0143-7)
Supplement: Supplementary file 2 — Table S2. Ophthalmological findings in healthy adult horses treated with tamoxifen. (PDF 30 kb) [file 13620_2019_143_MOESM2_ESM.pdf]

Table S2. Ophthalmological findings in healthy adult horses treated with tamoxifen (0.25 mg/kg, orally, q 24 hours for 7 days), with 7 days of follow-up after the treatment period (n=20).

**Visual Test**	**Presence**	**Abscence**
Threat response	20	0
Pupilar reflex	20	0
Palpebral reflex	20	0
Corneal Reflex	20	0
Dazzle reflex	20	0
Corneal transparency	20	0
Aqueous humor transparency	20	0
Crystalline transparency	20	0
Vitreus humor transparency	20	0
**Ocular Fundus**	**Normal**	**Abnormal**
Optic nerve	20	0
Retineal blood vessels	20	0
Non-tapetal fundus	20	0
No tapetal fundus	20	0
