# Supplementary material for: Tamoxifen in horses: pharmacokinetics and safety study
Source: Ir Vet J. 2019 Jun 20;72:5. doi: 10.1186/s13620-019-0143-7 (PMC6587269; doi:10.1186/s13620-019-0143-7)
Supplement: Supplementary file 3 — Table S1. LC–MS/MS conditions used for determination of TAM and its metabolites in equine plasma. (PDF 29 kb) [file 13620_2019_143_MOESM3_ESM.pdf]

Table S1. LC–MS/MS conditions used for determination of TAM and its metabolites in equine plasma.

Parameter	Setting
ESI	Positive mode
Capilar temperature	350 °C
Source collision induced dissociation	4 V
Tube lens voltages range	122 – 126 V
Voltaje spray	4 kV
Sheath gas pressure	60 psi
Auxiliary gas (Nitrogen)	10 AU
Q2 collision gas (Argon)	1.5 mTorr (0.2 Pa)
Q2 collision induced dissociation (CID)	10 V

ESI: Electrospray ionization
